# Modifiable risk factors for 9-year mortality in older English and Brazilian adults: The ELSA and SIGa-Bagé ageing cohorts

**DOI:** 10.1038/s41598-020-61127-7

**Published:** 2020-03-09

**Authors:** Marciane Kessler, Elaine Thumé, Shaun Scholes, Michael Marmot, Luiz Augusto Facchini, Bruno Pereira Nunes, Karla Pereira Machado, Mariangela Uhlmann Soares, Cesar de Oliveira

**Affiliations:** 1 Department of Postgraduate Program in Nursing, Federal University of Pelotas (UFPel), Pelotas, Rio Grande do Sul Brazil; 2 Department of Epidemiology & Public Health, University College London (UCL), London, United Kingdom; 3 Department of Postgraduate Program in Epidemiology, Federal University of Pelotas (UFPel), Pelotas, Rio Grande do Sul Brazil

**Keywords:** Lifestyle modification, Geriatrics, Health policy, Epidemiology, Risk factors

## Abstract

To quantify and compare 9-year all-cause mortality risk attributable to modifiable risk factors among older English and Brazilian adults. We used data for participants aged 60 years and older from the English Longitudinal Study of Ageing (ELSA) and the Bagé Cohort Study of Ageing (SIGa-Bagé). The five modifiable risk factors assessed at baseline were smoking, hypertension, diabetes, obesity and physical inactivity. Deaths were identified through linkage to mortality registers. For each risk factor, estimated all-cause mortality hazard ratios (HR) and population attributable fractions (PAF) were adjusted by age, sex, all other risk factors and socioeconomic position (wealth) using Cox proportional hazards modelling. We also quantified the risk factor adjusted wealth gradients in mortality, by age and sex. Among the participants, 659 (ELSA) and 638 (SIGa-Bagé) died during the 9-year follow-up. Mortality rates were higher in SIGa-Bagé. HRs and PAFs showed more similarities than differences, with physical inactivity (PAF 16.5% ELSA; 16.7% SIGa-Bagé) and current smoking (PAF 4.9% for both cohorts) having the strongest association. A clear graded relationship existed between the number of risk factors and subsequent mortality. Wealth gradients in mortality were apparent in both cohorts after full adjustment, especially among men aged 60–74 in ELSA. A different pattern was found among older women, especially in SIGa-Bagé. These findings call attention for the challenge to health systems to prevent and modify the major risk factors related to non-communicable diseases, especially physical inactivity and smoking. Furthermore, wealth inequalities in mortality persist among older adults.

## Introduction

Non-communicable diseases (NCDs) are the main cause of death worldwide, killing 41 million people annually, equivalent to 71% of all deaths globally^1^. The main groups of NCDs are cardiovascular diseases (17.9 million deaths), cancers (9.0 million deaths), respiratory diseases (3.9 million deaths), and diabetes (1.6 million deaths)^1^. NCDs are often associated with deaths at older age, but evidence shows that a considerable percentage of deaths due to NCDs are premature (before 70 years)^1^. The four NCDs listed above cause over 80% of premature deaths, with over 85% occurring in low- and middle-income countries^1^.

NCD-related health and lifestyle behaviours typically follow the social gradient: people who are disadvantaged in terms of socioeconomic position (SEP) have worse health – and live shorter lives – than those more advantaged^2^. Inequalities in social conditions have long-lasting effects during a lifetime that contribute to high levels of NCD-related risk factors^3,4^. Socioeconomically disadvantaged persons are at greater risk of being exposed to harmful products, such as tobacco, or of having unhealthy diets, and typically they have lower levels of access to healthcare services^1^. Having an unhealthy diet, being physically inactive, and being a cigarette smoker may result in raised blood pressure, increased blood glucose, elevated blood lipids and obesity; all modifiable risk factors associated with NCDs and greater risk of premature death^1^. Analyses of ageing cohorts typically show that social gradients in modifiable risk factors explain at least in part the observed social gradients in mortality at older ages^5,6^.

Most recent epidemiological evidence on the significant risk factors for NCDs and mortality in older adults comes from longitudinal studies conducted in mainly high-income countries (HICs) in Europe^7–12^, United States^13–15^, and Asia^16,17^. Longitudinal evidence on the associations between NCD-related risk factors and mortality among older persons is scarce in low-and middle-income countries (LMICs). However, a number of ageing cohorts began in LMICs in the 2000s; hence, a decade of follow-up now allows an opportunity to begin to fill this gap in the evidence. Furthermore, a detailed comparison of ageing cohorts between a HIC such as England and a LMIC such as Brazil provides an opportunity to broaden our understanding of the consequences of NCD-related risk factors and the impact of social inequalities in health in populations where the epidemiologic transition (i.e. the increase in incidence and mortality from NCDs) is more recent^18^ and which have the fastest growing ageing populations globally^4^.

A previous comparison of the mortality risks attributable to NCD-related risk factors used data from the English Longitudinal Study of Ageing (ELSA) and the Bambui Cohort Study of Ageing, based in Bambui city in the Minas Gerais State in Southeast region, located in the middle of Brazil^19^. In contrast to the Bambui Cohort, the Bagé Cohort Study of Ageing (SIGa-Bagé) enables us to observe the recent mortality experiences of older Brazilians in a very different social and economic context. SIGa-Bagé is the second population-based study to follow-up an ageing cohort in the South region of Brazil. Bagé city is located in South-western Brazil, in the State of Rio Grande do Sul (RS). Compared to Minas Gerais, levels of literacy, human development, per capita income and life expectancy (LE) at birth are higher in Rio Grande do Sul, whilst levels of infant mortality and vulnerability to poverty are lower^20^. On the other hand, all-cause and NCD-specific mortality rates among elderly persons are slightly higher^21^ due to higher LE in the South region.

No studies to date have quantified the mortality risks attributable to NCD-related risk factors in the SIGa-Bagé cohort. We used data from the ELSA and SIGa-Bagé cohorts to quantify and compare the 9-year all-cause mortality risks attributable to five modifiable risk factors (smoking, hypertension, diabetes, obesity and physical inactivity) among adults aged 60 years and older. In addition, we quantified and compared the social gradients in mortality after adjustment for all risk factors, stratified by age and sex.

## Results

From the 6015 (ELSA) and 1593 (SIGa-Bagé) eligible participants at baseline, complete risk factor and wealth data was available for 3315 and 1373 participants, respectively. Those included in our analyses were slightly younger than those excluded in ELSA (70.4 years [SD 7.7] vs. 72.8 years [SD 8.8]; *P* < 0.001), but were slightly older in SIGa-Bagé (71.6 years [SD 8.4] vs. 68.5 years [SD 6.6]; *P* < 0.001). Inclusion in the analytical sample showed no sex difference (*P* = 0.139 ELSA; *P* = 0.102 SIGa-Bagé), however, wealthier participants were most likely to have complete data (*P* < 0.001 ELSA; *P* = 0.056 SIGa-Bagé). ELSA participants with a favourable risk factor profile (i.e. non-smokers, non-obese, physically active, normotensive, and non-diabetic) were more likely to be included (all *P* < 0.005); but the chances of inclusion did not vary by risk factors in SIGa-Bagé (all *P* > 0.05).

For the two analytical samples, Table 1 shows selected baseline characteristics. Relative to ELSA, SIGa-Bagé participants were similar in age (mean: 70.4 and 71.6 years), but had a higher proportion of women (54.4% vs. 62.0%) and, as expected, a lower proportion in the richest wealth group (47.5% vs. 26.1%). Levels of current smoking, hypertension, diabetes and inactivity were higher in SIGa-Bagé, whilst obesity was lower (27.1% ELSA; 17.0% SIGa-Bagé). Overall, 78.1% and 82.5% of ELSA and SIGa-Bagé participants had at least 1 risk factor; 13.2% and 12.9% had 3+.

**Table 1:** Selected baseline characteristics of participants, by cohort (ELSA and SIGa-Bagé cohort studies).

Characteristics	ELSA (n = 3315)	SIGa-Bagé (n = 1373)
Mean age in years (SD)	70.4 (7.7)	71.6 (8.4)
Female (%)	54.4	62.0
Current smoking (%)	9.7	15.5
Physically inactive (%)	21.0	41.4
**Hypertension:**		
Diagnosed hypertension (%)	45.7	55.5
Mean SBP (mmHg)	137.2 (18.8)	—
Mean DBP (mmHg)	74.3 (10.9)	—
Survey hypertension/diagnosis (%)^b^	64.7	—
**Diabetes:**		
Diagnosed diabetes (%)	8.6	15.3
HbA1c ≥ 6.5%	7.5	—
Survey diabetes (%)^b^	10.7	—
**BMI:**		
Mean BMI (kg/m^2^) (SD)	27.6 (4.6)	26.0 (4.4)
Non-obese (%)^a^	73.0	83.0
Obese (%)	27.1	17.0
**Number of risk factors**^**b**^ **(%):**		
None	21.9	17.5
One	39.2	38.3
Two	25.7	31.2
Three or more	13.2	12.9
**Wealth (%):**		
Poorest	31.2	34.8
Middle	21.2	39.1
Richest	47.5	26.1

BMI: body mass index; DBP: diastolic blood pressure; SBP: systolic blood pressure; SD: standard deviation

^a^Excluded 29 underweight (<18.5) in England and 28 in Brazil.

^b^Smoking, hypertension (ELSA: survey-defined), diabetes (ELSA: survey-defined), obesity, and physical inactivity.

**Figure 1:**
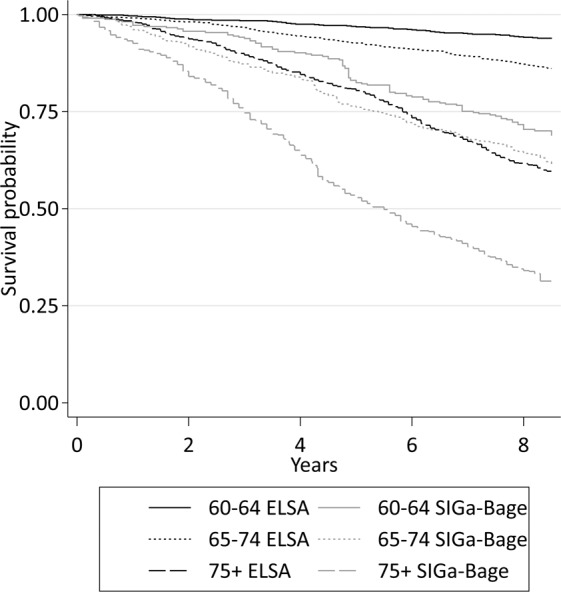
Nine-year sex-adjusted survival probability among participants in the English (ELSA) and Brazilian (Bagé) ageing cohorts by age group.

Overall, 659 deaths occurred in ELSA over a mean duration of follow-up of 7.8 years (SD 1.8); 638 deaths occurred in SIGa-Bagé over 6.4 years (SD 2.6). The unadjusted mortality rate was approximately 2.8 times higher in SIGa-Bagé than in ELSA (72.3 and 25.4 deaths per 1,000 person-years, respectively). Figure 1 displays the cohort specific sex-adjusted KM curves showing the proportion of members surviving over the 9-year follow-up by age group. Relative to ELSA, survival probabilities were lower in the SIGa-Bagé cohort at each age, with the survival gap being widest among those 75+.

Overall and separately for each risk factor, Table 2 shows the cohort-specific mortality rates (unadjusted) and three sets of HRs corresponding to *Model 1* (age- and sex-adjusted), *Model 2* (Model 1 + other risk factors), and *Model 3* (Model 2 + wealth). The estimates changed little after wealth-adjustment: therefore, for the purposes of brevity, we summarise results for *Model 3* only. In fully adjusted analyses, physical inactivity was a strong predictor of subsequent mortality in both cohorts (ELSA: HR = 1.75, *P* < 0.001; SIGa-Bagé: HR = 1.50, *P* < 0.001), as was current smoking (ELSA: HR = 1.66, *P* < 0.001; SIGa-Bagé: HR = 1.45, *P* = 0.005). The diabetes HR was similar in magnitude in both cohorts, but only attained statistical significance in ELSA (ELSA: HR = 1.28, *P* = 0.032; SIGa-Bagé: HR = 1.23, *P* = 0.087). Obesity did not attain significance in either cohort; whilst hypertension only attained marginal significance in SIGa-Bagé (SIGa-Bagé: HR = 1.18; *P* = 0.073).

**Table 2:** Mortality rates and hazard ratios for over a 9-year follow-up period among English and Brazilian ageing cohorts, by selected baseline risk factors (The ELSA and SIGa-Bagé cohort studies).

	Deaths (rate per 1000 person years)		Model 1^a^: HR (95% CI)		Model 2^b^: HR (95% CI)		Model 3^c^: HR (95% CI)	
	ELSA	SIGa-Bagé	ELSA	SIGa-Bagé	ELSA	SIGa-Bagé	ELSA	SIGa-Bagé
All	659 (25.4)	638 (72.3)	—	—	—	—	—	—
**Smoking:**								
No	575 (24.5)	532 (71.1)	1	1	1	1	1	1
Yes	84 (34.6)	105 (78.3)	1.96 (1.55–2.48)	1.40 (1.12–1.76)	1.73 (1.35–2.23)	1.43 (1.10–1.85)	1.66 (1.29–2.14)	1.45 (1.12–1.89)
			P < 0.001	P = 0.004	P < 0.001	P = 0.008	P < 0.001	P = 0.005
**Hypertension:**								
No	184 (19.8)	281 (71.8)	1	1	1	1	1	1
Yes	475 (28.6)	357 (72.7)	1.14 (0.95–1.36)	1.10 (0.94–1.28)	1.05 (0.88–1.26)	1.19 (0.99–1.43)	1.04 (0.87–1.25)	1.18 (0.98–1.42)
			P = 0.148	P = 0.246	P = 0.575	P = 0.055	P = 0.666	P = 0.073
**Diabetes:**								
No	560 (24.0)	534 (71.0)	1	1	1	1	1	1
Yes	99 (37.4)	104 (79.8)	1.45 (1.17–1.80)	1.19 (0.97–1.47)	1.30 (1.04–1.63)	1.24 (0.98–1.56)	1.28 (1.02–1.60)	1.23 (0.97–1.55)
			P = 0.001	P = 0.102	P = 0.023	P = 0.075	P = 0.032	P = 0.087
**Obesity:**								
No	465 (24.8)	433 (69.4)	1	1	1	1	1	1
Yes	179 (25.5)	77 (58.6)	1.25 (1.05–1.48)	1.07 (0.84–1.36)	1.13 (0.95–1.35)	1.04 (0.82–1.33)	1.10 (0.92–1.32)	1.05 (0.82–1.34)
			P = 0.011	P = 0.592	P = 0.181	P = 0.724	P = 0.314	P = 0.699
**Inactivity:**								
No	404 (19.2)	300 (53.4)	1	1	1	1	1	1
Yes	254 (51.6)	338 (105.2)	1.96 (1.65–2.32)	1.63 (1.39–1.92)	1.80 (1.51–2.14)	1.52 (1.27–1.82)	1.75 (1.47–2.09)	1.50 (1.25–1.79)
			P < 0.001	P < 0.001	P < 0.001	P < 0.001	P < 0.001	P < 0.001
**Risk Factors:**								
None	82 (14.0)	78 (56.6)	1	1	—	—	1	1
One or more	561 (28.3)	431 (69.8)	1.60 (1.26–2.03)	1.35 (1.07–1.72)	—	—	1.52 (1.20–1.92)	1.35 (1.06–1.71)
			P < 0.001	P = 0.012	—	—	P = 0.001	P = 0.014
One	206 (20.1)	181 (60.3)	1.17 (0.90–1.51)	1.11 (0.86–1.45)	—	—	1.15 (0.89–1.49)	1.11 (0.86–1.44)
Two	234 (37.1)	169 (74.1)	1.99 (1.54–2.58)	1.51 (1.15–1.98)	—	—	1.90 (1.46–2.46)	1.50 (1.15–1.97)
Three or more	121 (36.8)	81 (91.0)	2.23 (1.69–2.96)	1.91 (1.40–2.61)	—	—	2.06 (1.54–2.75)	1.88 (1.37–2.57)
			P < 0.001*	P < 0.001*	—	—	P < 0.001*	P < 0.001
*Trend*			1.37 (1.26–1.49)	1.27 (1.16–1.39)	—	—	1.33 (1.22–1.45)	1.26 (1.16–1.38)
			P < 0.001**	P < 0.001**	—	—	P < 0.001**	P < 0.001**

^*a*^*Model 1* (age- and sex-adjusted); ^b^*Model 2* (Model 1 + other risk factors); ^c^*Model 3* (Model 2 + wealth as a categorical variable); * Joint test; **Test for trend (number of risk factors entered as a single continuous variable).

Compared to those with none, having 1 or more risk factors was a strong predictor of subsequent mortality in both cohorts (ELSA: HR = 1.52, *P* = 0.001; SIGa-Bagé: HR = 1.35, *P* = 0.014). Furthermore, a significant graded association between the number of risk factors and mortality was evident in both cohorts. For example, compared to those with none, the age-, sex-, and wealth-adjusted HRs for participants with 3 + risks was 2.06 (*P* < 0.001 for the joint Wald test) in ELSA and 1.88 (*P* < 0.001) in SIGa-Bagé (Table 2).

### Mortality attributable to risk factors (PAF)

**Figure 2:**
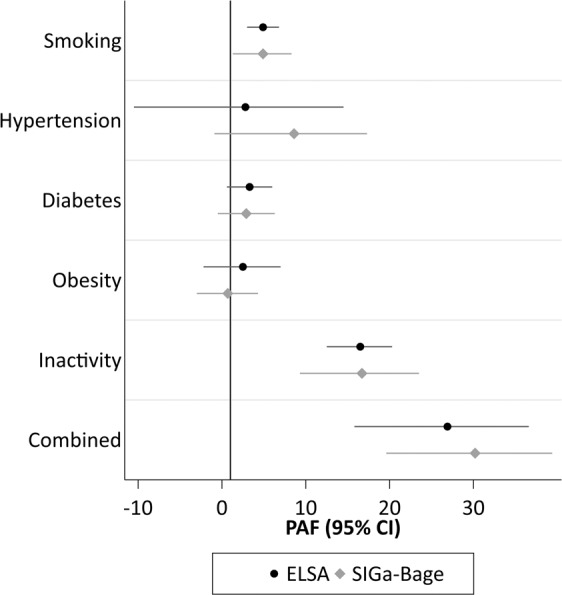
PAFs for all-cause mortality among participants in the English (ELSA) and Brazilian (Bagé) ageing cohorts.

Supplementary Table 1 shows the cohort-specific PAFs for mortality separately for each risk factor and for the combined PAF. Results changed little after wealth-adjustment; therefore, we summarise results for *Model 3* only (adjustment for age, sex, all other risk factors and wealth). Figure 2 displays the estimated PAFs. Consistent with the HRs upon which the PAFs were calculated, the fully-adjusted PAFs were largest in magnitude in both cohorts for inactivity (ELSA: PAF 16.5%: 95% CI: 12.4, 20.3%; SIGa-Bagé: PAF 16.7%: 95% CI: 9.3, 23.5%) and for current smoking (ELSA: PAF 4.9%: 95% CI: 3.0, 6.8%; SIGa-Bagé: PAF 4.9%: 95% CI: 1.3, 8.3%). Results for diabetes attained significance in ELSA (PAF 3.3%: 95% CI: 0.6, 6.0%) but not in SIGa-Bagé (PAF 2.9%: 95% CI: −0.5, 6.3%). The estimated combined PAF - comparing the “baseline” scenario (observed prevalence of each risk factor at baseline) with the “fantasy” scenario (prevalence of *each* risk factor set to zero) - was similar in magnitude for both cohorts (ELSA: PAF 26.9%: 95% CI: 15.8, 36.6%; SIGa-Bagé: PAF 30.2%: 95% CI: 19.6, 39.4%).

### Mortality by wealth

**Figure 3:**
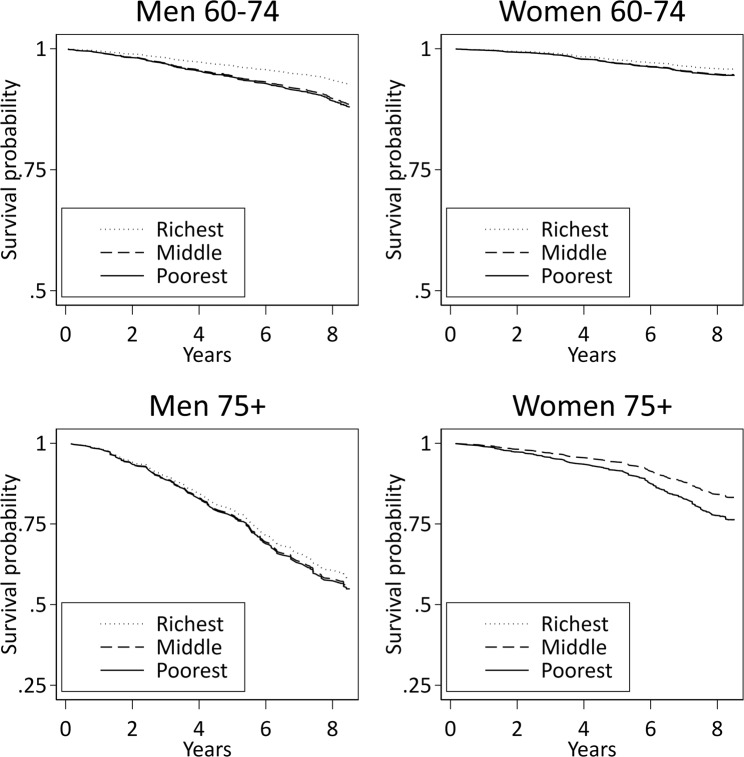
Social gradient in all-cause mortality after adjustment for risk factors by age and sex. ELSA.

**Figure 4:**
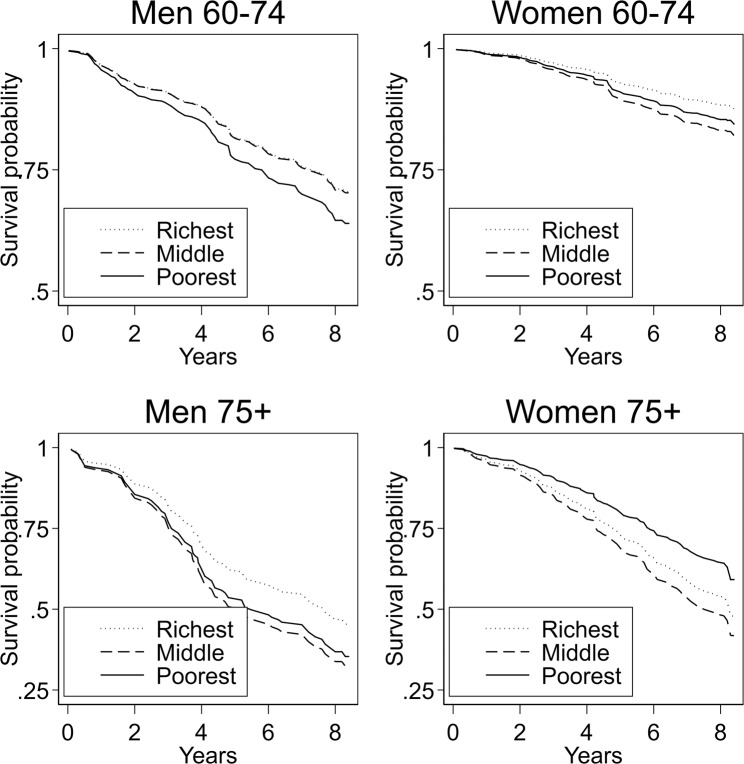
Social gradient in all-cause mortality after adjustment for risk factors by age and sex. SIGa-Bagé.

Figure 3 (ELSA) and Figure 4 (SIGa-Bagé) show the risk-factor adjusted wealth-gradients in all-cause mortality by age (60–74; 75+) and sex. Mortality was higher in all groups in SIGa-Bagé relative to ELSA, mainly at older ages. Wealth gradients in mortality were slightly evident among men (younger and older ages) and among younger women in both cohorts, but did not attain significance after full adjustment, except among men aged 60–74 in ELSA (*p* = 0.012 for the joint Wald test). The pattern was different among women aged 75+; suggesting higher mortality in the middle wealth group in ELSA, but which did not attain significance after full adjustment. In contrast, mortality among older women in SIGa-Bagé appeared to be higher among the richest relative to those in the poorest and middle wealth groups (*p* = 0.036 for the joint Wald test) (Supplementary Table 2).

## Discussion

In the present study, we quantified and compared the 9-year all-cause mortality risk attributable to five modifiable NCD-related risk factors – current smoking, hypertension, diabetes, high BMI and physical inactivity – among older English and Brazilian adults. Independently of sex, the all-cause mortality risk was 2.8 times higher among participants in the Brazilian cohort (SIGa-Bagé) at all ages. However, the risk factor and subsequent mortality associations generally showed more similarities than differences. In both cohorts, inactivity and smoking were the strongest risk factors in fully-adjusted analyses; and our results showed a clear graded relationship between the number of risk factors (at baseline) and mortality. Wealth gradients in mortality were slightly evident in both cohorts, attaining significance in fully-adjusted analyses among men aged 60–74 in ELSA. A different pattern was found among older women, especially in SIGa-Bagé.

The higher mortality risk for SIGa-Bagé participants reflects at least in part the differences in sociodemographic profile between the two ageing cohorts. 47.5% of ELSA participants were in the richest wealth group, compared to 26.1% in SIGa-Bagé. Our estimates from the ELSA cohort should be interpreted in light of selective loss to follow-up (known as the “healthy survivor effect”) as those in our analytical sample took part at Wave 2 and had complete risk factor and wealth data. Notwithstanding differences in risk factor definitions (due to the reliance on self-reported data in SIGa-Bagé), levels of current smoking, hypertension, diabetes and inactivity were higher in SIGa-Bagé, but obesity prevalence was lower. Our finding corroborates with a previous comparison of Brazilian and English ageing cohorts with the former based on the Bambui study^19^.

In addition to differences in socio-demographic profile and risk factor prevalence, the differences in mortality risk between the two ageing cohorts reflect differences in social contexts and healthcare service provision. Life expectancy (LE) at birth is currently 75.8 and 81.2 years in Brazil and the UK respectively; LE at age 65 is 19.8 and 21.1 years^22^. Gross domestic product (GDP) is around 15.7 and 44.9 thousand dollars/capita in Brazil and the UK respectively^23^, with average annual wages (in thousand dollars in 2017) being around 7.9 in Brazil^24^ and 44.6 in England^25^. Brazil and England both have universal health systems, with medical and nurse care accessible to all, and have a system of primary care that is not available in many countries^26–29^. Notwithstanding the universal coverage, Brazil spends far less on health than England, as shown by numerous indicators. For example, the per capita health expenditure is US $995 and $4192 in Brazil and in England, respectively; whilst the proportion of GDP spent on health is 3.9% and 7.6%, respectively. On the other hand, 25% of the Brazilian population is covered by private health insurance compared to 11% in England: trends in coverage from 2005 to 2015 show an increase in Brazil and a decrease in England. Likewise, the number of practising doctors (1.8 and 2.8 per 1000 population) and nurses (1.5 and 7.9) is lower in Brazil than in England^22^. Health gains in both countries may be compromised by the recent - and future - economic recessions. In Brazil, the National Basic Health Care Policy (PNAB) has been revised^30^ and austerity policies involving a long-term freeze on public expenditure has been approved^29,31^. UK governments have also implemented austerity measures over the last decade, particularly affecting the most vulnerable^32^.

Although we compare the mortality experience of older English and Brazilian adults in the present study, it is imperative to highlight the fact that Brazil is a large and heterogeneous country with regards to social and economic development, as well as culture. Such heterogeneity is also manifested in its wide spectrum of health outcomes varying from infection diseases and external causes to chronic and non-communicable diseases^19,33,34^. In 2016, the socio-demographic index (a composite average of the income per capita, average educational attainment and fertility rate that classifies countries or geographic areas’ level of development), LE at birth and healthy LE were higher in the Southeast and South regions compared to the North and Northeast regions, whilst the age-standardised disability-adjusted life-years (DALYs) rate was lower^34^. The social inequality observed between geographical regions and states in Brazil inevitably leads to health inequities, as evidenced recently^31,33,34^. The burden of disease and higher mortality are generally higher in the states of the North and Northeast regions compared to the South and Southeast^33,34^. In addition, the disease profile varies among the regions. Evidence suggests that states in the South and Southeast of Brazil are in the later stages of the epidemiological transition towards NCDs compared to states in the North and Northeast^34^, due, at least in part, to higher LE and faster population ageing^20^. On the other hand, states in the earlier stages of the epidemiological transition are facing a double burden of communicable, maternal, neonatal, and nutritional diseases and non-communicable diseases, alongside a growing burden related to external causes across Brazil^34^.

### Modifiable risk factors and mortality

Of the five NCD-related modifiable risk factors, physical inactivity and current smoking were most strongly associated with subsequent mortality in both cohorts. This agrees with findings from similar studies in other ageing cohorts in the UK, Brazil and elsewhere^8,11,19,35,36^. Worldwide, physical inactivity and smoking are among the major NCD-related risk factors, considered preventable diseases^1^. According to the Global Burden of Disease (GBD), the median percentage change in deaths attributable to low physical activity was 46.5% from 1990 to 2013^37^ and was 22% from 2007 to 2017^38^.

In our study, 21% and 41% of ELSA and SIGa-Bagé participants were classed at baseline as being physically inactive. The fully-adjusted HRs for inactivity were 1.75 and 1.50 in ELSA and SIGa-Bagé respectively; with the PAFs showing a hypothetical (elimination of inactivity) proportional reduction in mortality of around 17% in both cohorts. Longitudinal evidence on the inactivity-mortality associations is scarce; but English and Brazilian studies have shown that being physically active is associated with reduced mortality risk, and that lower levels of activity increase the risk^11,35,36^. The precise mechanisms by which inactivity increases mortality risk are yet to be fully determined. Being physically active is linked to favourable biomarker profiles such as lower fat mass and decreased adipose tissue inflammation^39^ and conferring a reduced risk of mental and physical limitations or disabilities, and other preventable NCDs^40,41^. On the other hand, it is important to bear in mind that reverse causality could also explain, at least in part, these associations, since participants classified as physically inactive may have been unable to engage in regular physical activity due to ill health^42^.

Tobacco use remains a major determinant of global health, ranking second overall (behind high SBP) in terms of risk to death and to DALYs worldwide, and being the leading risk factor in HICs, including those in Western Europe^38,43^. The number of deaths attributable to tobacco smoking increased from 4.6 million in 1990 to 5.8 million in 2013^37^ and stood at 7.1 million in 2017, amounting to an increase of 24·9% (95% CI: 21·3–28·6) from 1990 to 2017^38^. In the present study, 9.7% and 15.5% of ELSA and SIGa-Bagé participants respectively were current smokers at baseline. The fully-adjusted estimated HRs for smoking were 1.66 and 1.45 in ELSA and SIGa-Bagé respectively; with the PAFs showing a hypothetical (elimination of smoking) proportional reduction in mortality of around 5% in both cohorts.

Our findings agree with a previous Brazilian and English comparison based on the Bambui Study and ELSA^19^. A recent literature review of longitudinal studies on the smoking-mortality association in community-dwelling older adults concluded that there was a scarcity of studies from LICs; smoking was associated with mortality in all studies, reinforcing smoking as an established risk factor for premature mortality. The HRs comparing current- and non-smokers have ranged from 1.3 to 5.0; with the highest HRs observed in HICs^12,13,16,19,44–46^. In addition to cardiovascular disease (CVD)-specific mortality, smoking is a major risk factor for several types of cancer^46^, reflecting its broad impact on all-cause mortality, and its strong association with premature death^12^.

Diabetes prevalence was higher in the SIGa-Bagé cohort (15.3% self-reported diagnosed) compared to ELSA (10.7% self-reported diagnosed and/or HbA1c ≥ 6.5%); and was a significant risk factor for subsequent mortality in ELSA, but only attained borderline significance in SIGa-Bagé. Based on the estimated PAFs, a mortality reduction of around 3% could be achieved through the elimination of diabetes. Our findings agree with a previous Brazilian (9% in the Bambui study) and ELSA (5%) comparison^19^. Data from the Bambui study was collected between 1997 and 2002; since 2003, more effective public health policies towards diabetes prevention and control have been implemented at the national level (including the Family Health Strategy [FHS]). Although the number of diabetes-related deaths increased in Brazil between 1990 and 2015, the corresponding increase in age-standardized mortality rates did not attain significance^33^.

Hypertension was the most prevalent risk factor in ELSA (64.7% diagnosed and/or ≥140/90 mmHg) and in SIGa-Bagé (55.5% diagnosed). However, the fully-adjusted HRs did not attain significance in ELSA, and it achieved only borderline significance in SIGa-Bagé. In agreement with the study by de Oliveira *et al*.^19^, a proportional reduction of mortality of around 9% (SIGa-Bagé) and 3% (ELSA) could hypothetically be achieved through elimination of hypertension. Notwithstanding the relatively weak hypertension-mortality association observed in the present study, the higher PAF in SIGa-Bagé possibly mirrors the fact that ischaemic heart disease (IHD) and cerebrovascular diseases are the leading causes of death at the state and national levels^33^. Evidence suggests that the rates of these diseases fell from 1990 to 2015^33^, with possible explanations for this decline including better pre-hospital and hospital care for IHD- and stroke-patients^33^, and improvements in cardiovascular health at the population level – including better control of hypertension and diabetes - through the expansion of the FHS and other factors^27^.

In the present study, obesity was the only risk factor with higher prevalence in ELSA (27.1% versus 17% in SIGa-Bagé): though we acknowledge that BMI was measured objectively (through height and weight measurements) in ELSA whilst being self-reported in SIGa-Bagé. It is well known that reliance on self-reports of height and weight lead to an underestimation of obesity prevalence^47^. In both cohorts, the fully-adjusted HRs for obesity failed to attain significance, and the PAFs were below 1%. Comparing estimates of the PAF for the obesity-mortality associations across studies are problematic due to practical issues such as the definition of obesity, the choice of an appropriate counterfactual exposure, the choice of appropriate relative risks, the interpretation of the results, and inconsistencies/inaccuracies in the estimation of PAFs^48^.

A recent study confirmed BMI as a poor predictor of mortality among ELSA cohort members^49^; and that being obese conferred no excess risk compared to having a normal weight^49^. Results have been similar in other ageing cohorts, with the term “obesity paradox” referring to the counterintuitive findings reported elsewhere of a survival advantage in older age among those obese^50^. In contrast, being underweight and/or experiencing weight loss have been shown to confer excess mortality risk among older adults in Brazil, UK and elsewhere^44,49,51,52^.

Finally, a significant graded association between the number of risk factors and mortality was evident in both cohorts. The combined PAFs based on the assumption that all risk factors were simultaneously eliminated and that the effects of the risk factors on the hazard function were multiplicative (additive on the log scale) - meaning that the combined effect of the risk factors equalled the product of their separate effects – showed a hypothetical proportional reduction in mortality of around 30% in both cohorts^53^. Our findings agree with previous evidence using data from the ELSA and Bambui cohorts^19^.

### Socioeconomic gradients in mortality

Our analysis suggests higher mortality in both cohorts among the poorest men relative to the richest, although significance after risk factor adjustment was only attained among men aged 60–74 in ELSA. This finding is consistent with previous studies that found wider social inequalities in all-cause mortality among men than in women^54^; a finding which could be explained at least in part by gender differences in behavioural (e.g. smoking and alcohol consumption) and psychosocial factors^55^. Other ELSA studies have also shown that the strength of the association between wealth and all-cause mortality was stronger among younger participants^56^.

However, our study also showed an absence of a social gradient in all-cause mortality among women aged 60–74 in both cohorts. Among women aged 75+, we similarly found no social gradient in both cohorts, with the 9-year mortality risk appearing to be higher in the richest SIGa-Bagé participants relative to the poorest. This unexpected result contrasts with previous evidence from Brazil, where social inequalities in mortality were observed for men and women aged 60–79, although inequalities were not observed among women aged 80+^57^. In a North American study, the all-cause mortality rate was higher among African Americans than among Whites, Asian/Pacific Islanders, or Hispanics, but at older ages, the racial and ethnic differences in death rates narrowed^58^, suggesting an age effect. Our finding may also be explained at least in part by a healthy survivor bias, where those recruited to the SIGa-Bagé study from the poorest groups may have been in better health on average.

In general, our results show that adjustment for the five modifiable risk factors considered in our study could explain the absence of a wealth gradient in all-cause mortality in most groups. In previous ELSA studies, wealth was associated with all-cause mortality, with a pronounced social gradient at younger and older ages, even after adjustment for health behaviours^56,59^. More studies are needed to understand the magnitude of the social gradients in all-cause mortality in the SIGa-Bagé cohort, taking into account the high levels of social inequality and the diversity of health outcomes in the city. Also in this context, it is important to highlight that at the time of data collection for the baseline study, a strong primary health care model – including the FHS – covered the most deprived areas in Bagé, but it was not available in the less deprived areas. Being enrolled in the FHS has been shown to have significantly narrowed the gap in health service access, usual source of care^27,28,60^, healthcare outcomes and mortality^61,62^ between the richest and the poorest (and/or racial) groups. Expenditure and sufficient public financing on health and social protection has therefore seemed to mitigate some of the detrimental health effects of living in the most deprived areas, especially among vulnerable populations, helping to reduce health inequalities^29,63^. Future studies of the SIGa-Bagé cohort and other national longitudinal studies should therefore consider the role of the FHS in potentially modifying the wealth gradients in all-cause mortality and promotion of health equity among older adults.

### Strengths and limitations

The main strength of our study was the comparison between two ageing cohorts from countries with different social and economic contexts. Whilst ELSA data has been extensively used in the epidemiological literature, this is the first study to use the SIGa-Bagé cohort. Other strengths include a higher number of modifiable risk factors than previous studies, and the lengthier follow-up (nine-years) compared to other studies^19^. Both cohorts had comparable procedures for ascertaining mortality with minimal losses to follow-up. This enabled us to accurately estimate the HRs and PAFs for 9-year mortality due to five major NCD-related risk factors in two very different ageing cohorts, adding to previous cross-national comparisons.

There were a number of important limitations. Mortality data (time of death) was incomplete for a small number of SIGa-Bagé participants; but the imputation of survival time for 59 participants would have had only a modest impact on our findings. Both the risk factor and wealth variables were assessed at baseline and, therefore, we did not capture any change in these variables over time. Thus, the risk factor and mortality associations may be underestimated to some extent due to regression dilution bias (i.e. imprecise measurement of the exposure variables). In comparison to the wide range of health examination data available in ELSA, objective measures of height and weight, glycated haemoglobin, and blood pressure were not available in SIGa-Bagé. For this reason, we decided a priori not to perform statistical tests to formally compare mortality risks between the two cohorts. We also decided a priori to provide information (although measured differently) on all five risk factors rather than limit the study to the subset of risk factors that were comparable. Our findings for the SIGa-Bagé cohort need to be interpreted in light of the potential misclassification of diabetes, hypertension and obesity through the use of self-reported data. We recommended that objective measures should be included in future SIGa-Bagé data collection efforts. SIGa-Bagé data were collected in Rio Grande do Sul state, a relatively wealthy state in the South region. However, the data collection took place in a city situated in the poorest area of the state^20^, highlighting the relevance of this study, as the Brazilian states are themselves very heterogeneous. We acknowledge that there are other Brazilian ageing cohorts, such as The Brazilian Longitudinal Study of Aging (ELSI-Brazil)^64^. In comparison to the SIGa-Bagé cohort, cohorts such as ELSI-Brazil^64^ may have a wider geographical coverage (and so be more nationally-representative) and have superior risk factor measurement that may be better suited for future cross-national comparative studies. At the moment, however, only cross-sectional data is available for ELSI-Brazil. Finally, our PAF estimates should be interpreted in light of the assumptions made in the calculations: namely, that the risk factor and subsequent mortality association is causal, meaning that the mortality risks will change as predicted, if we intervene by achieving zero risk factor prevalence^65^.

In conclusion, smoking and physical inactivity were found to be the main risk factors of all-cause mortality among older English and Brazilian adults, suggesting that both factors should remain a key focus of health policy debates, to prevent NCDs and subsequent mortality risk. Older adults should be encouraged to increase their levels of physical activity and to quit smoking, considering previous evidence about the benefits of smoking cessation and physical activity on mortality even later in life^13,36^. Furthermore, diabetes was found to be a risk factor of all-cause mortality over the 9-year follow-up period, mainly among older English adults. Despite the efforts made worldwide to improve diabetes prevention and treatment, further improvement is still needed. This finding highlights the challenge imposed to health systems^19^.

Social gradients in mortality should be addressed, mainly in low and middle-income countries, however, more comparative studies are needed to quantify the magnitude of social gradients in mortality at older ages, and to provide evidence on the specific role of primary health care in reducing social inequalities. Our results provide some support to the hypothesis that most predictors of mortality in middle-to-older age do not vary across populations.

## Methods

### English longitudinal study of ageing (ELSA)

ELSA is a large-scale, ongoing prospective ageing cohort study. The baseline sample (2002–03) comprised 11,391 participants aged 50 years and older living in private households. ELSA data allows exploration of the dynamic relationships between health, functioning, social networks and economic position. Participants have follow-up interviews every 2-years, and health examinations every 4-years. ELSA’s methodology is described in detail elsewhere^66^. The baseline analytical sample in the present study comprised persons aged 60 and over who took part in the second wave (2003–04) as this coincided with the first collection of health examination data. Ethical approval for ELSA was obtained from the London Multicentre Research Ethics Committee. Participants gave full informed consent to taking part in the study and for linkage to mortality data. All methods were performed in accordance with relevant guidelines and regulations.

### Bage cohort study of ageing (SIGa-Bagé)

SIGa-Bagé was designed and developed to investigate the incidence and predictors of adverse health outcomes in an elderly Brazilian population, where most residents were classified in the lowest income, wealth and educational groups. SIGa-Bagé is one of the first longitudinal study of Brazilian older persons that proposed to assess the impact of Primary Health Care services on social inequities in health and specifically in premature mortality. Sampling design and data collection methods of the study are described elsewhere^60^. All participants aged 60 and over living in private households interviewed at the baseline interview in 2008 (n = 1,593) were eligible for this analysis. Surviving members completed face-to-face interviews after a period of 8 to 9 years. The SIGa-Bagé study was approved by the Ethics Board of the Federal University of Pelotas, Rio Grande do Sul, Brazil. All participants gave informed consent and all methods were performed in accordance with relevant guidelines and regulations.

### Mortality ascertainment

Consenting ELSA participants were linked to the National Health Service’s Central Registry, which provides vital status data. 659 deaths occurring from Wave 2 (2003–04) to 2013 were included in the analysis, ensuring a similar length of follow-up to that for SIGa-Bagé. Deaths in SIGa-Bagé were reported to the study team in person or by phone by the next of kin during the follow-up interview and were ascertained through the Brazilian System of Information on Mortality. Death certificates were obtained for 91% of the participants at baseline who were reported to have died. For the minority of participants (n = 59) with no death certificate (and so no exact date of death was available) we imputed the missing data using the sex- and age-specific mean survival time to avoid loss of data.

### NCD risk factors

We decided a priori to focus on five leading modifiable NCD-related risk factors: current smoking, hypertension, diabetes, high body mass index (BMI), and physical inactivity. These risk factors were defined using the most complete data available for each cohort.

#### ELSA

Current cigarette smokers were identified based on self-report. Participants were asked questions on self-reported doctor-diagnosis for hypertension and diabetes. Using measurements obtained using standardised protocols, we calculated levels of systolic (SBP) and diastolic blood pressure (DBP) as the mean of the second- and third-measurements. Glycated haemoglobin (HbA1c) was measured using blood samples. Hypertension was defined as a previous medical diagnosis for the disease and/or SBP ≥ 140 mmHg and/or DBP ≥ 90 mmHg. Diabetes was defined as a previous medical diagnosis and/or HbA1c ≥ 6.5%. Obesity was defined as BMI ≥ 30.0 kg/m^2^, based on measured height and weight^67^. Underweight participants (BMI < 18.5 kg/m^2^; *n* = 29) were excluded due to small numbers and likely confounding with disease/ill-health. Participants were asked how often they engaged in moderate and in vigorous sports/activities; we classed participants as inactive if they did not take part in either moderate or vigorous activities at least once a week.

#### SIGa-Bagé

Current smokers were identified based on self-report. Information on hypertension and diabetes was based on self-reported doctor-diagnosis (i.e. “*Did a doctor ever tell you that you had…?*”). BMI was measured using self-reported weight and height, with obesity defined as BMI ≥ 30.0 kg/m^2^: 28 underweight participants were excluded for the same reason as described for ELSA. Participants were classed as physically inactive if they neither walked nor did any moderate- or vigorous-intensity activities for at least 10 minutes at least once a week.

### Demographics and socioeconomic position

In both studies, age (Wave 2 in ELSA; baseline in SIGa-Bagé) was classified into three groups: 60–64; 65–74; 75+. We used wealth as the primary measure of socioeconomic position (SEP) following previous research based on the ELSA cohort^56^ which showed that wealth was more strongly associated with mortality than other measures of SEP such as educational attainment and occupational class^56^. In ELSA, total household wealth (net of debt) at Wave 2, including financial wealth (savings and investments), the value of any home and other property (less mortgage), business assets, and physical wealth was divided into age-adjusted quintiles (based on the whole sample). These were merged in the present study into three groups: poorest (quintiles 1 and 2); middle (quintile 3); and richest (quintiles 4 and 5). Wealth was assessed in SIGa-Bagé using the Brazilian Economic Classification Criteria. This utilises information on house furniture(s), car(s), housekeeper(s) and the highest educational qualification of the head of household. Participants were originally grouped into five categories (A richest; E poorest)^68^; these were merged in our study into three groups: poorest (D/E); middle (C); and richest (A/B).

### Statistical analyses

Four sets of analyses were performed separately in each cohort. First, to examine differences in age-specific mortality, sex-adjusted cumulative survival curves were computed using the Kaplan-Meier (KM) method. Second, to examine the associations between NCD risk factors and all-cause mortality, we used Cox proportional hazards (PH) models (after confirming non-violation of the PH assumption through examination of Schoenfeld residuals). Results from sequential models were summarised using hazard ratios (HR). In *Model 1*, we examined the associations for each risk factor in isolation after sex- and age-adjustment. In *Model 2*, we examined the associations for each risk factor whilst additionally adjusting for the other risk factors (i.e. Model 1 + other risk factors). We investigated potential interactions between risk factors and mortality by wealth (entered into the models as a categorical variable) but there were none. Therefore, in *Model 3*, we examined the wealth-adjusted associations for each risk factor (i.e. Model 2 + wealth). In separate analyses, we ran each of these models using a variable which classed participants as not having or having at least one risk factor; and using a categorical variable based on the number of risk factors (0, 1, 2, and 3+). We also entered the number of risk factors as a continuous variable (range 0 to 5) and used the p-value as a test for trend.

Third, for *Models 1–3*, we used the estimates from the Cox model to calculate the corresponding population attributable fraction (PAF) separately for each risk factor. PAFs were calculated using the *punafcc* command in Stata which was specifically developed for time-to-event studies and which implements the method recommended by Greenland and Drescher^69^; this estimates the log of the mean hazard ratio between two scenarios: a “*baseline*” scenario (risk factor prevalence as observed at baseline), and a “*fantasy*” scenario, in which the prevalence of the risk factor in question was set to zero (with all other variables taking the same values as the baseline scenario). This ratio is known as the population unattributable fraction (PUF), and the PUF is subtracted from 1 to obtain the PAF, which can be interpreted as the proportional reduction in mortality that would occur if the risk factor prevalence at baseline was reduced to zero^65^. The magnitude of the PAF is dependent on the strength of the effect of a risk factor on mortality, the absolute risk of mortality, as well as the prevalence of the risk factor in the study population^65^. PAFs were computed using the HRs from the three sequential models described above. In addition, we computed a combined PAF comparing the “baseline” scenario (risk factor prevalence as observed) with a “fantasy” scenario where the prevalence of *each* risk factor was set to zero.

Fourth, the risk factor-adjusted wealth gradients in all-cause mortality were quantified using HRs. The models were stratified by age-group (60–74; 75+) and sex due to observed differences in the wealth-mortality associations. Statistical significance of the wealth gradients in mortality were examined using a joint Wald test (wealth entered as a three-category variable; richest as reference) and by a test for trend (wealth entered as a single continuous variable). All analyses were conducted in Stata V15.0 (StataCorp, College Station, Texas). All p-values were two-tailed and statistical significance was defined as *p* < 0.05.

## Supplementary information

Supplementary Table.

## Data Availability

All data generated or analysed during this study are included in this published article (and its Supplementary Information Files).
