# Supplementary material for: Modifiable risk factors for 9-year mortality in older English and Brazilian adults: The ELSA and SIGa-Bagé ageing cohorts
Source: Sci Rep. 2020 Mar 9;10:4375. doi: 10.1038/s41598-020-61127-7 (PMC7062886; doi:10.1038/s41598-020-61127-7)
Supplement: Supplementary file 1 — Supplementary Table. [file 41598_2020_61127_MOESM1_ESM.pdf]

Modifiable risk factors for 9-year mortality in older English and Brazilian adults: The ELSA and SIGa-Bagé ageing cohorts

Marciane Kessler[1,2]*, Elaine Thumé[1], Shaun Scholes[2], Michael Marmot[2], Luiz Augusto Facchini[1,3], Bruno Pereira Nunes[1], Karla Pereira Machado[1], Mariangela Uhlmann Soares[1], Cesar de Oliveira[2]

Supplementary Table 1. Population attributable fractions (PAF) for 9-year mortality by selected baseline risk factors (The ELSA and SIGa-Bagé cohort studies)

	**Model 1 [a]**		**Model 2 [b]**		**Model 3 [c]**	
	**PAF % (95 CI)**	**P**	**PAF % (95 CI)**	**P**	**PAF % (95 CI)**	**P**
**ELSA**						
Smoking	6.3 (3.6, 8.8)	**<0.001**	5.2 (3.4, 7.0)	**<0.001**	4.9 (3.0, 6.8)	**<0.001**
Hypertension	8.8 (-3.4, 19.6)	0.152	3.6 (-9.5, 15.2)	0.576	2.8 (-10.5, 14.5)	0.664
Diabetes	4.7 (1.7, 7.5)	**0.002**	3.6 (0.8, 6.2)	**0.010**	3.3 (0.6, 6.0)	**0.017**
Obesity	5.6 (1.1, 9.8)	**0.015**	3.2 (-1.3, 7.5)	0.161	2.5 (-2.2, 7.0)	0.298
Inactivity	18.9 (14.0, 23.5)	**<0.001**	17.0 (13.2, 20.7)	**<0.001**	16.5 (12.5, 20.3)	**<0.001**
Combined*	-	-	28.9 (18.3, 38.1)	**<0.001**	26.9 (15.8, 36.6)	**<0.001**
**SIGa-Bagé**	**PAF % (95 CI)**	**P**	**PAF % (95 CI)**	**P**	**PAF % (95 CI)**	**P**
Smoking	4.7 (1.4, 7.8)	**0.005**	4.6 (1.0, 8.1)	**0.011**	4.9 (1.3; 8.3)	**0.008**
Hypertension	4.9 (-3.6, 12.8)	0.248	9.1 (-0.3, 17.7)	0.058	8.6 (-0.9; 17.3)	0.075
Diabetes	2.6 (-0.6, 5.7)	0.110	3.0 (-0.4, 6.4)	0.086	2.9 (-0.5; 6.3)	0.098
Obesity	0.9 (-2.6, 4.4)	0.597	0.6 (-3.0, 4.1)	0.725	0.7 (-3.0; 4.3)	0.701
Inactivity	20.5 (14.0, 26.5)	**<0.001**	17.3 (9.9, 24.0)	**<0.001**	16.7 (9.3; 23.5)	**<0.001**
Combined*	-	-	30.9 (20.4, 39.9)	**<0.001**	30.2 (19.6; 39.4)	**<0.001**

PAF: Population Attributable Fraction;
[a] **Model 1** (age- and sex-adjusted); [b] **Model 2** (Model 1 + other risk factors); [c] **Model 3** (Model 2 + wealth as a categorical variable). For each risk factor, the PAF compares the "baseline" scenario (risk factor prevalence as observed) with a "fantasy" scenario where the prevalence of the risk factor in question was set to zero.
*Combined PAF compares the "baseline" scenario (risk factor prevalence as observed) with a "fantasy" scenario where the prevalence of **each** risk factor was set to zero.

Supplementary Table 2. Mortality rates and hazard ratios by wealth for nine-year mortality among English and Brazilians by wealth (The ELSA and SIGa-Bagé cohort studies.

	**Deaths (rate per 1000 person years)**		**HR (95% CI)[a]**		**Deaths (rate per 1000 person years)**		**HR (95% CI)[a]**	
	**Age 60-74**				**Age 75+**			
	**ELSA**	**SIGa-Bagé**	**ELSA**	**SIGa-Bagé**	**ELSA**	**SIGa-Bagé**	**ELSA**	**SIGa-Bagé**
**Wealth Men**								
Poorest	62 (26.7)	55 (78.7)	**1.69 (1.15-2.48)**	1.28 (0.81-2.00)	74 (85.5)	44 (171.5)	1.11 (0.79-1.55)	1.31 (0.77-2.25)
Middle	39 (22.2)	52 (54.8)	**1.61 (1.07-2.42)**	1.01 (0.66-1.54)	47 (81.4)	45 (146.2)	1.09 (0.76-1.56)	1.43 (0.83-2.45)
Richest	59 (12.5)	44 (56.4)	1.0	1.0	85 (68.2)	22 (104.6)	1.0	1.0
Trend **			**1.30 (1.08-1.56)**	1.13 (0.89-1.42)			1.05 (0.89-1.25)	1.12 (0.87-1.45)
**Wealth Women**								
Poorest	45 (14.2)	60 (45.3)	1.32 (0.82-2.11)	1.28 (0.79-2.06	82 (56.5)	77 (101.0)	1.00 (0.70-1.41)	0.71 (0.44-1.15)
Middle	23 (10.2)	73 (48.2)	1.28 (0.75-2.18)	1.49 (0.96-2.33)	32 (34.8)	86 (145.5)	0.68 (0.44-1.03)	1.18 (0.73-1.89)
Richest	38 (7.6)	34 (31.7)	1.0	1.0	72 (47.6)	38 (122.1)	1.0	1.0
Trend **			1.15 (0.91-1.44)	1.11 (0.89-1.38)			1.00 (0.83-1.21)	0.81 (0.65-1.02)

[a] Adjusted for age+ risk factors; **Test for trend (wealth entered as a single continuous variable). Statistically significant coefficients shown in bold.
